# Estimation abilities of large numerosities in Kindergartners

**DOI:** 10.3389/fpsyg.2013.00518

**Published:** 2013-08-29

**Authors:** Sandrine Mejias, Christine Schiltz

**Affiliations:** Educational Measurement and Applied Cognitive Science, Université du LuxembourgWalferdange, Luxembourg

**Keywords:** approximate number system, early number competence, numeracy, estimation, non-symbolic numbers, symbolic numbers, socio-economic factors, mathematical development

## Abstract

The approximate number system (ANS) is thought to be a building block for the elaboration of formal mathematics. However, little is known about how this core system develops and if it can be influenced by external factors at a young age (before the child enters formal numeracy education). The purpose of this study was to examine numerical magnitude representations of 5–6 year old children at 2 different moments of Kindergarten considering children's early number competence as well as schools' socio-economic index (SEI). This study investigated estimation abilities of large numerosities using symbolic and non-symbolic output formats (8–64). In addition, we assessed symbolic and non-symbolic early number competence (1–12) at the end of the 2nd (*N* = 42) and the 3rd (*N* = 32) Kindergarten grade. By letting children freely produce estimates we observed surprising estimation abilities at a very young age (from 5 year on) extending far beyond children's symbolic explicit knowledge. Moreover, the time of testing has an impact on the ANS accuracy since 3rd Kindergarteners were more precise in both estimation tasks. Additionally, children who presented better exact symbolic knowledge were also those with the most refined ANS. However, this was true only for 3rd Kindergarteners who were a few months from receiving math instructions. In a similar vein, higher SEI positively impacted only the oldest children's estimation abilities whereas it played a role for exact early number competences already in 2nd and 3rd graders. Our results support the view that approximate numerical representations are linked to exact number competence in young children before the start of formal math education and might thus serve as building blocks for mathematical knowledge. Since this core number system was also sensitive to external components such as the SEI this implies that it can most probably be targeted and refined through specific educational strategies from preschool on.

## Introduction

Math abilities are of fundamental importance in modern society and possessing good mathematical knowledge critically determines the likelihood of employment (e.g., Rivera-Batiz, 1992). Yet we are unfortunately not all equal in learning math: Some of us excel in the mathematical domain and dedicate their careers to it while others struggle with in school and later avoid it at any cost. But even before formal math education has started young children do not enter school with the same chances. Especially pupils from low socio-economic families seem to be at risk for mathematical failure and a difference in early number skills was already noticed in preschoolers (e.g., Jordan et al., 2006) which was then evolving toward a global mathematics underachievement in middle to high school students (Dossey et al., 1988).

In all these cases, however, math ability is thought to develop based on the Approximate Number System (ANS), an ontogenetically and phylogenetically primitive system dedicated to numerosity processing (Cantlon, 2012). The ANS is known to develop throughout the lifespan. Yet, how factors such as education and socio-economic environment influence this development are fundamental questions that still need to be fully elucidated. To what extend ANS serves as a building block for arithmetical knowledge and supports procedures for numerical computation is a related issue. Indeed, understanding the basis of typical development will help us developing good educational strategies, identifying the deficits observed in mathematical learning difficulties and dyscalculia and elaborating evidence-based guidelines for remediation.

Up to now, three categories of behavioral tasks have been used to assess approximate number representations in animals and humans: estimation, comparison, and approximate calculation. Performance in those tasks is supposed to index specifically the memory^1^ representations of the analog quantity system and is thought to reflect the quality of an individual's ANS. Several regularities across the different types of comparison, approximate calculation, or estimation tasks could be singled out.

*What are the signatures of the ANS and its development?* In *comparison* (i.e., choose the largest numerosity) and *approximate calculation tasks* (i.e., add or subtract two large numerosities and compare the resulting sum to a third numerosity), participants' performance depends on the numerical ratio between the non-symbolic stimuli. This corresponds to a limit of the system, which can be measured through the Weber fraction (*W*), an important signature of the ANS. Up to now research in typical development has consistently revealed that the critical discrimination ratio narrows with age, i.e., the ability to discriminate between two numerosities improved with age (see Halberda et al., 2012, for the reverse trend in elderly). This ability, already present a few hours after birth (Izard et al., 2009), allows infants to discriminate the numerosity of small sets of objects (e.g., Starkey and Cooper, 1980), or even larger ones when the ratio between them is large enough (e.g., Xu and Spelke, 2000). A developmental increase in precision was reported by Piazza et al. (2010) using a classical comparison task of two dot sets in 5 and 10 year old children. Mundy and Gilmore (2009) also showed this increase in another comparison paradigm: the children had to map a symbolic target (i.e., Arabic symbols presented with pre-recorded number words) with one of the two alternative non-symbolic choices (dot sets of 20–50 dots) or they had to do the reverse mapping (map a non-symbolic target numerosity with one of the two alternative symbolic choices). The authors observed a performance increase between 6 and 8 years of age with generally better results for the mapping from dots to Arabic numbers than for the reverse.

Performance in *estimation tasks* (i.e., freely produce a symbolic or non-symbolic equivalent of the numerosity) confirm that, non-human species (e.g., Platt and Johnson, 1971) and humans are able to process numerical quantities approximately, no matter what the modality/format of the input and output are (non-symbolic to symbolic visual or verbal, Whalen et al., 1999; Castronovo and Seron, 2007 or the reverse symbolic to non-symbolic mapping process, Whalen et al., 1999; Cordes et al., 2001). In these tasks, over- and underestimation errors are observed, which seem to depend on the direction of the mapping, in the sense that overestimations are associated with symbolic to non-symbolic mappings, whereas non-symbolic to symbolic mapping is related to underestimations (see for example Castronovo and Seron, 2007; Crollen and Seron, 2012). Moreover, the estimations of the target magnitudes are generally inaccurate such that mean estimates and response variability both increase with target magnitude, indicating that the underlying representation is less precise for larger numerosities. More specifically, this representation is characterized by a scalar variability which gives rise to a constant coefficient of variation (COV = standard deviation of mean response/mean response) across target magnitudes (Whalen et al., 1999; Cordes et al., 2001; Mejias et al., 2012a,b; Castronovo and Göbel, 2012). Over early development, the variability of the representations decreases with age while their precision increases. Indeed, studies of typical development have consistently reported increasing precision of the ANS with age. In a study by Huntley-Fenner's (2001), 5–7 year olds had to estimate the numerosity of a set of black squares (5–11 items) on a number line consisting of a series of Arabic numbers ordered from 0 to 20. Mean accuracy significantly increased throughout the age range of 5–7 years and COV scores were negatively correlated with age in days (COVs ranged from 0.37 to 0.11), showing that estimates were less variable with increasing age. In an estimation study using larger numerosities, Mejias et al. (2012b) reported that mean 9 year olds' COV was 0.29, which is higher than adult's mean COV (0.16 in Mejias et al., 2012a). The precision and the variability of this approximate analog representation consequently seem to be related to development (see also Chillier, 2002; Booth and Siegler, 2006).

*How do ANS refinements relate to math achievement?* Several studies directly investigated the link between children's ANS and their abilities in learning numbers symbols and arithmetic. Since study outcomes were quite divergent, it is not yet clear how the interactions between ANS and symbolic number knowledge arise and develop. The conflicting outcomes could have different origins because different age-ranges of populations have been tested with different types of tasks (i.e., comparison, approximate calculations, and estimation as detailed above) probing ANS.

In Piazza and collaborators' study (2010), 5–10 year old children's non-symbolic number acuity did not correlate with (symbolic) arithmetical scores (for similar results in 4–7 year olds see Soltesz et al., 2010). In Mundy and Gilmore's study with 6–8 year olds, scores on the mapping tasks did not correlate with arithmetical scores. Congruent with those results, the school mathematics performance of 6–8 year old children was found to be unrelated to the magnitude of their numerical distance effect exhibited in a comparison task involving non-symbolic numerical displays (Holloway and Ansari, 2009; Sasanguie et al., 2013). Such a relation was on the contrary obtained on a similar comparison task with symbolic numbers in 6 year olds, since children with a smaller symbolic distance effect showed higher mathematics performance (De Smedt et al., 2009; Holloway and Ansari, 2009).

Yet, others did, however, report that western adolescents' ANS precision was clearly related to performance in exact calculation and number processing. They observed a correlation between the accuracy in a non-symbolic numerical comparison task performed at age 14 and school mathematics performance from Kindergarten to sixth grade (Halberda et al., 2008). Gilmore et al. (2010) also provide evidence that the ANS precision might be related to symbolic knowledge. Based on studies of Barth et al. (2005, 2006) they evaluated 5–6 year old children during their first year of school and found that children's performance on large-number non-symbolic approximate addition related significantly to their mastery of school's mathematics curriculum at the end of that first year of formal instruction (e.g., counting objects, recognizing Arabic digits, symbolic and non-symbolic comparisons—all numerical tasks using numbers smaller than 10). It appeared that non-symbolic arithmetic performance was related to children's mathematics achievement 3 months later, independently of achievement levels in reading or intelligence and socio-economic background. Congruently, Mussolin et al. (2012) examined the performance of 3 different groups of children from Kindergarten to grade 1. When considering them together, a positive relation between their accuracy in discriminating sets of non-symbolic elements and their ability to process numerical symbols was observed even when taking intelligence and short-term memory into account (see also Libertus et al., 2011). Finally, data collected from Amazonian Munduruku indigene children and adults show that Munduruku with a certain level of symbolic number knowledge have a more refined ANS than their peers with little or no formal instruction (Pica et al., 2004). Similarly, it was recently reported that math education sharpens the approximate numerical representations in western adults (Nys et al., 2013).

*In summary*, the ANS seems to serve representing the approximate cardinal values of large sets of stimuli and it can be assessed using estimation, comparison and approximate calculation tasks. Parallel signatures of the ANS were found in studies of human adults, children, infants (and even non-human animals). They provide evidence for the existence of a magnitude-based estimation system for representing symbolic and non-symbolic numerical magnitude that also supports procedures for numerical computation, even outside formal education. According to some authors (e.g., Barth et al., 2005), this is congruent with the fact that the ANS serves as building block for symbolic arithmetic learning. However, currently this assumption is also challenged by several studies, which consistently failed to observe a correlation between non-symbolic numerical magnitude comparison and mathematical performance at the beginning of formal school education (e.g., Holloway and Ansari, 2009; Mundy and Gilmore, 2009). To resolve this contradiction and better understand the observed changes in ANS acuity, further studies investigating young children's relation between ANS and exact number knowledge are urgently required.

In the present study, we evaluated preschool children's ability to estimate large non-symbolic numerosities. Children had to produce estimates of large numerosities ranging from 8 to 64 elements, i.e., clearly exceeding number values included in their school curricula^2^. Estimation abilities for these large quantities were assessed via both non-symbolic (i.e., 64 differently sized elements) and symbolic (i.e., “64”) output formats. To the best of our knowledge there are currently no studies investigating large quantity estimation in preschoolers. The rational was to assess and compare both symbolic as well as non-symbolic estimation abilities of large numerosities (and the underlying ANS representations) in preschool children, i.e., before these numerosities are systematically learned and their exact meaning is mastered. To highlight the influence of preschool math education on estimation abilities we compared children from the 2nd and the 3rd Kindergarten grade while they were performing symbolic and non-symbolic estimation. In addition children's early number competence levels associated with the two Kindergarten grades were evaluated using exact number processing tasks involving numerosities up to 12 items. To analyze the effect of environmental influences such as the socio-economic status on approximate (and exact) number abilities in preschool children, we compared the performances of children coming from two schools characterized by different levels of socio-economic index (SEI). Finally, we used an individual differences approach to investigate whether preschoolers' early (symbolic and non-symbolic) number competence might relate to their accuracy in these approximate numerical tasks.

**Figure 1:**
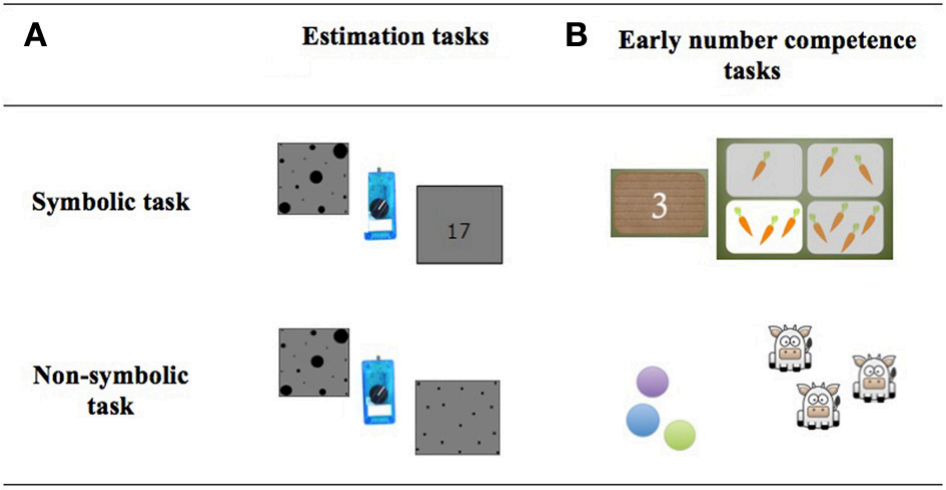
**Symbolic and non-symbolic tasks in the (A) estimation and (B) the early number competence tasks**.

Because our design included both symbolic and non-symbolic tasks we could systematically investigate the relationship between exact and approximate numerical abilities for these two task formats (see Figure 1). While others have evaluated either symbolic (i.e., Gilmore et al., 2007; Mundy and Gilmore, 2009) or non-symbolic (i.e., Gilmore et al., 2010) approximate number abilities, there are currently no studies evaluating the two types of approximate processing within the same population of preschool children (see (Mejias et al., 2012b) for this type of evaluation in 9–10 year old 4th graders). This seems, however, particularly important given the above-mentioned controversies concerning the role of non-symbolic vs. symbolic number abilities as precursors of math performance (e.g., Halberda et al., 2008 vs. De Smedt et al., 2009; Holloway and Ansari, 2009; Gilmore et al., 2010). Indeed it is complicated to compare the correlations between approximate numerical abilities and math competence observed in different studies since they might be confounded by subtle differences related to group (e.g., age, environmental context) or study design (e.g., math test battery).

In addition we believe that the free estimation tasks used in the present study are more sensitive to individual differences in numerical processing than the predominantly used comparison tasks. First, the comparison paradigms might lack sensitivity because they assess performance for a limited number of predefined numerical ratios (e.g., Rousselle and Noël, 2007; De Smedt and Gilmore, 2011). Under those conditions, it is always possible to miss a significant difference between participant groups if the ratios selected are not sensitive enough. Second, it was recently argued that the mechanisms used to extract information from dot-arrays in comparison tasks is driven by visual features rather than numerical dimensions (Szucs et al., 2013). In contrast, numerical estimation tasks cannot be solved by only relying on perceptual processes since they typically require producing estimation outputs in a different format than the input (e.g., heterogeneous to homogeneous dots, dots to Arabic digits, dots to number words). Consequently we preferred to use a free production paradigm in which children have to produce a certain magnitude. This allowed us to measure directly the precision of the children's estimates. Accordingly, we hoped to unveil the so far controversial relationship between the ANS and children's early number competence.

We hypothesized that preschoolers would show the ANS signature when producing symbolic and non-symbolic estimation outputs for numbers largely exceeding their curricular pre-mathematical knowledge. Moreover, 3rd grade preschool children were expected to be more accurate than their peers from the 2nd grade. Especially the non-symbolic tasks assessing approximate and exact numerical abilities were expected to co-vary from an early age, whereas a certain level of number symbol mastery might be required before exact number symbols are linked to ANS. Concerning the impact of school's SEI on estimation abilities, predictions were less clear-cut given the mixed evidence in the literature (Ramani and Siegler, 2008; Gilmore et al., 2010). Yet in any case, it is critical to identify if and when external factors such as SEI impact estimation abilities (and early number competence) in order to optimally design and plan educational intervention.

## Methods

### Participants

Participants were 74 children coming from two different Belgium public Kindergarten schools. Parental consent was obtained for each of the children. One school was ranked as a school with a low SEI^3^ whereas the other was a middle SEI school. In each school, one group of children was tested at the end of the second grade (4–5 year olds, mid-June 2012) and another group at the end of the third grade (5–6 year olds, mid-June 2012). Children's descriptive information, according to the Kindergarten class they belonged to and their school's SEI, are presented in Table 1A.

**Table 1:** **(A) Descriptive information; (B) means (*SD*) of children's precision of numerical estimation calculated as an Absolute Error Score (AES; computed as: |the child's estimate answers—target magnitude|) by task for the different groups of children according the time of testing and the school SEI; (C) means (*SD*) of children's number of corrects trials by task for the different groups of children according the time of testing and the school SEI**.

**Time of testing—*School SEI***	**2nd grade—*low***	**2nd grade—*middle***	**3rd grade—*low***	**3rd grade—*middle***
**(A) DESCRIPTIVE INFORMATION**				
*N* (boys)	20 (10)	22 (11)	14 (5)	18 (9)
Age in months (*SD*)	59.15 (3.92)	57.82 (3.66)	71.79 (5.18)	70.78 (3.21)
**(B) ESTIMATION TASKS**				
Symbolic estimation task (*SD*)	71.08 (47.50)	53.39 (35.44)	45.64 (40.60)	32.23 (21.30)
Non-symbolic estimation task (*SD*)	52.08 (41.75)	37.62 (27.11)	35.08 (26.92)	15.40 (5.46)
**(C) EARLY NUMBER COMPETENCE TASKS**				
Symbolic association task (*SD*)	4.45 (2.35)	5.96 (3.89)	9.50 (2.88)	10.72 (1.27)
Non-symbolic trade task (*SD*)	5.20 (3.17)	6.23 (2.40)	9.36 (2.53)	10.67 (1.78)
Total early number competence score (*SD*)	9.65 (4.72)	12.18 (5.74)	18.86 (5.07)	21.39 (2.66)

Children who took part in the study had no history of developmental disorders and were considered as typically developing children by the Belgian psycho-medico-social services.

### Materials and procedure

#### Estimation tasks

Two computerized estimation tasks developed by Mejias et al. (2012b) were used to evaluate the children's ability to estimate large quantities. They took place on a PC-compatible portable (screen size: 30.5 × 23 cm) running E-Prime software (Schneider et al., 2002). Children sat about 55 cm away from the computer screen and had to estimate the numerosities of black dots displayed for 1 s on a gray screen in two tasks: (1) In the *symbolic estimation task*, children were presented with a set of heterogeneously-sized dots. They were asked to estimate the cardinality of each set by producing the corresponding Arabic number (AN, Arial font with a visual angle of 2.2°) using a potentiometer. In each set the size of the dots was manipulated so the total covered area was identical. However, to avoid larger collections also being those with smaller elements, dots of the smallest and largest size (respectively, with visual angle of 0.44 and 0.88°) were included in all sets. (2) In the *non-symbolic estimation task*, the children were presented with the same kind of sets of dots of mixed sizes but had to produce a collection of approximately the same number of equally sized dots (see Figure 1A).

Four numerosities were presented (8, 16, 34, and 64) six times to the children, providing a total of 24 stimuli for each task. Four practice trials by task were proposed to the children to familiarize themselves with the experimental setting on other numerosities (15, 25, 50, 75). In this training session, the participants received feedback (on the computer screen) corresponding to the correct answer, in order to allow a calibration of their estimation (Izard and Dehaene, 2008). Data from these trials are not reported in the analyses.

#### Early number competence tasks

To assess counting development, two tasks were administered to each child individually.

1. The symbolic *association task* tested the children's knowledge of Arabic digit symbols from 1 to 12 with their associated quantities. This task was administered in three steps starting with one card (A4 format) representing four sets of vegetables (from 1 to 4), followed by another card representing 4 other sets of vegetables (from 5 to 8) and the last card with 4 larger numerosities (from 9 to 12). Children were asked to associate the set of four small cards showing Arabic digits (from 1 to 4, 5 to 8, and 9 to 12 for the three steps, respectively) with the “right quantity of vegetables.” Trials were administered in a non-consecutive order within each step until the child made errors on three consecutive numerosities. The “association score” correspond to the highest number of correct association succeeded by the child.
2. The non-symbolic *trade task* assessed the non-verbal understanding of the one-to-one correspondence principles and was similar to the one used by Rousselle and Noël (2008). Children were given tokens while the experimenter presented them toy animals. In this context they were then invited to buy animals with their tokens: “You have coins and I have animals to sell. You can buy ONE animal by giving me ONE coin, no matter the size of the coin.” Two practice trials ensued in which the young participant was encouraged to exchange one large and then one small coin for one animal. Those practice trials were done to make sure that the children understood that coins' size did not matter. Then, three blocks were administered: starting with block A using numerosities from 1 to 4, then block B with numerosities from 5 to 8, and finally block C with numerosities ranging from 9 to 12. Numerosities inside a block were presented in a non-consecutive order, e.g., 2, 1, 4, and 3. Children were allowed to perform the task by giving one token for one animal or by counting. The task was interrupted if the child made errors on three consecutive numerosities (e.g., if a child failed on 5, 6, and 7). The “trade score” corresponded to the highest number of animals correctly traded (in this e.g., the trade score will be 4; see Figure 1B).

## Results

### Estimation tasks

#### The signatures of the approximate number system

**Figure 2:**
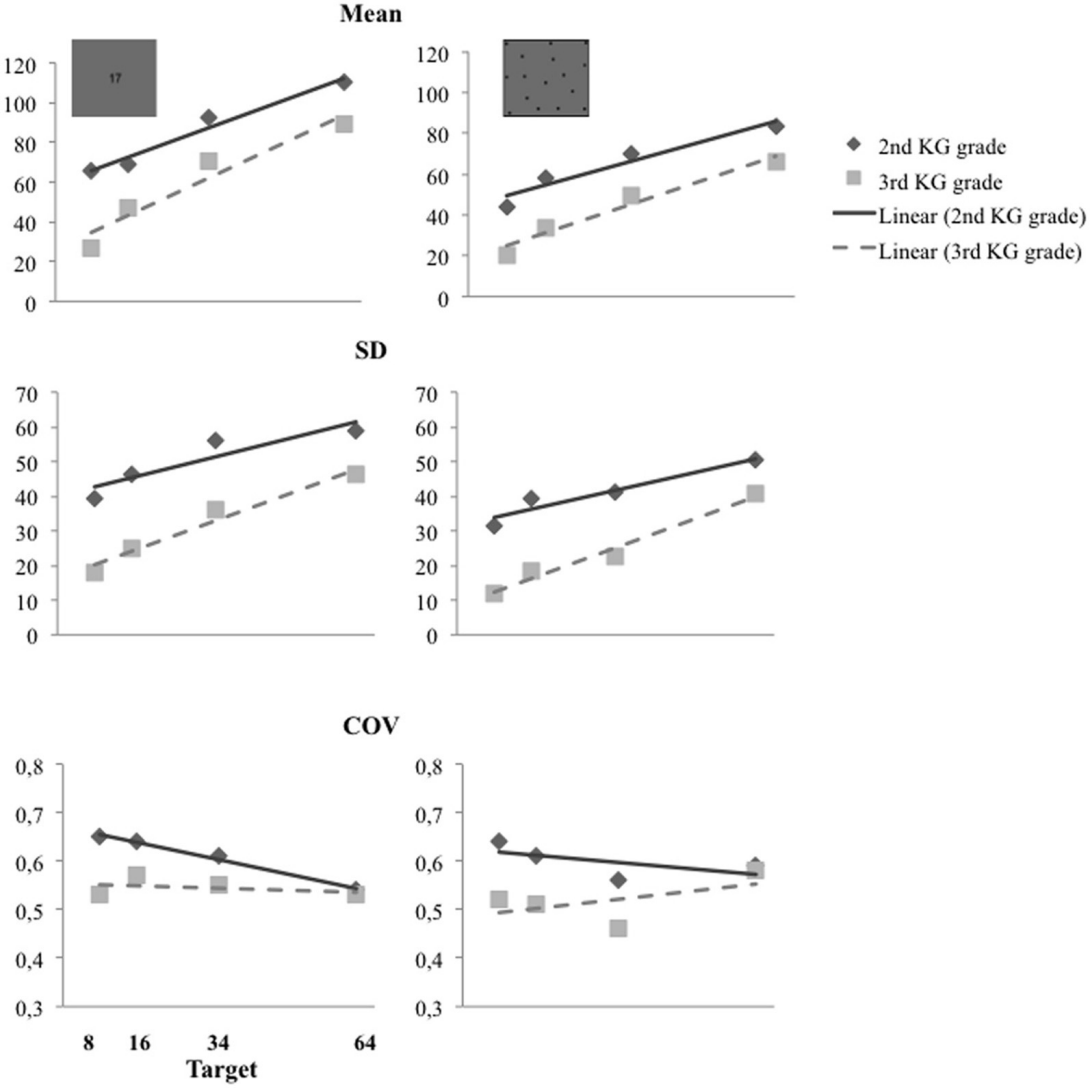
**Children's mean estimates and standard deviation (SD) increased linearly with the target magnitude in the symbolic and the non-symbolic estimation tasks.** The coefficients of variation (COV)—the ratios of the standard deviation to the mean—were approximately constant across the target magnitudes in both groups.

We first examined if preschool children showed the typical signatures of the ANS. All children's mean estimates and standard deviations (SD) increased in direct proportion to the target magnitudes while the coefficients of variation (COV; i.e., the ratio of the standard deviation to the mean estimate) remained constant across targets (Figure 2). In the non-symbolic and the symbolic estimation tasks the slopes of the mean estimates and their standard deviations were close to 1 (see Tables 2A,B), confirming the linear increase with the target size, sign of a typical numerical magnitude representation (e.g., Crollen et al., 2011; Mejias et al., 2012b). Moreover, as measured by the COV (see Table 2C), the variability of estimates was relative to target size in the two grade groups and in both estimation tasks: The slope of the best linear fit to the mean COV scores did not differ from 0 (*p*s > 0.1), except for the 2nd Kindergarten-graders in the symbolic “Dots to AN” task (children showed less variability in their answer for largest magnitudes to be estimated). The COV ranged from 0.31 to 0.89, with an average value of. 58 (±0.13) and from 0.20 to 0.92, with an average value of.56 (±0.17) in the present population of 5–6 year old children, respectively for the symbolic and the non-symbolic tasks. This provides direct evidence for scalar variability in preschool children's representation of numerosity in both tasks.

**Table 2:** **Results of the linear regression between the predictor variable (target results) and (A) the mean of the estimates, (B) the standard deviations of the estimates, and (C) the coefficients of variation (COV) of the estimates in the two estimation tasks for the two grades tested**.

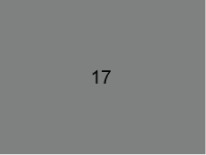		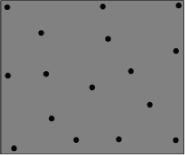	
**2nd KG grade**	**3rd KG grade**	**2nd KG grade**	**3rd KG grade**
**(A) MEAN OF ESTIMATES**			
*r*^2^ = 0.972	*r*^2^ = 0.927	*r*^2^ = 0.929	*r*^2^ = 0.955
β_0_ = 0.986	β_0_ = 0.963	β_0_ = 0.964	β_0_ = 0.977
*t*_(2)_ = 8.391^*^	*t*_(2)_ = 5.048^*^	*t*_(2)_ = 5.126^*^	*t*_(2)_ = 6.525^*^
**(B) STANDARD DEVIATION**			
*r*^2^ = 0.839	*r*^2^ = 0.958	*r*^2^ = 0.907	*r*^2^ = 0.978
β_0_ = 0.916	β_0_ = 0.979	β_0_ = 0.952	β_0_ = 0.989
*t*_(2)_ = 3.231^*^	*t*_(2)_ = 6.755^*^	*t*_(2)_ = 4.416^*^	*t*_(2)_ = 9.435^*^
**(C) COV**			
*r*^2^ = 0.988	*r*^2^ = 0.038	*r*^2^ = 0.295	*r*^2^ = 0.293
β_0_ = −0.994	β_0_ = −0.196	β_0_ = −0.543	β_0_ = 0.541
*t*_(2)_ = −13.029^**^	*t*_(2)_ = −2.830	*t*_(2)_ = −0.914	*t*_(2)_ = 0.910

* Correlation significantly different from 0 at p 0.05;

** at p 0.01.

**Figure 3:**
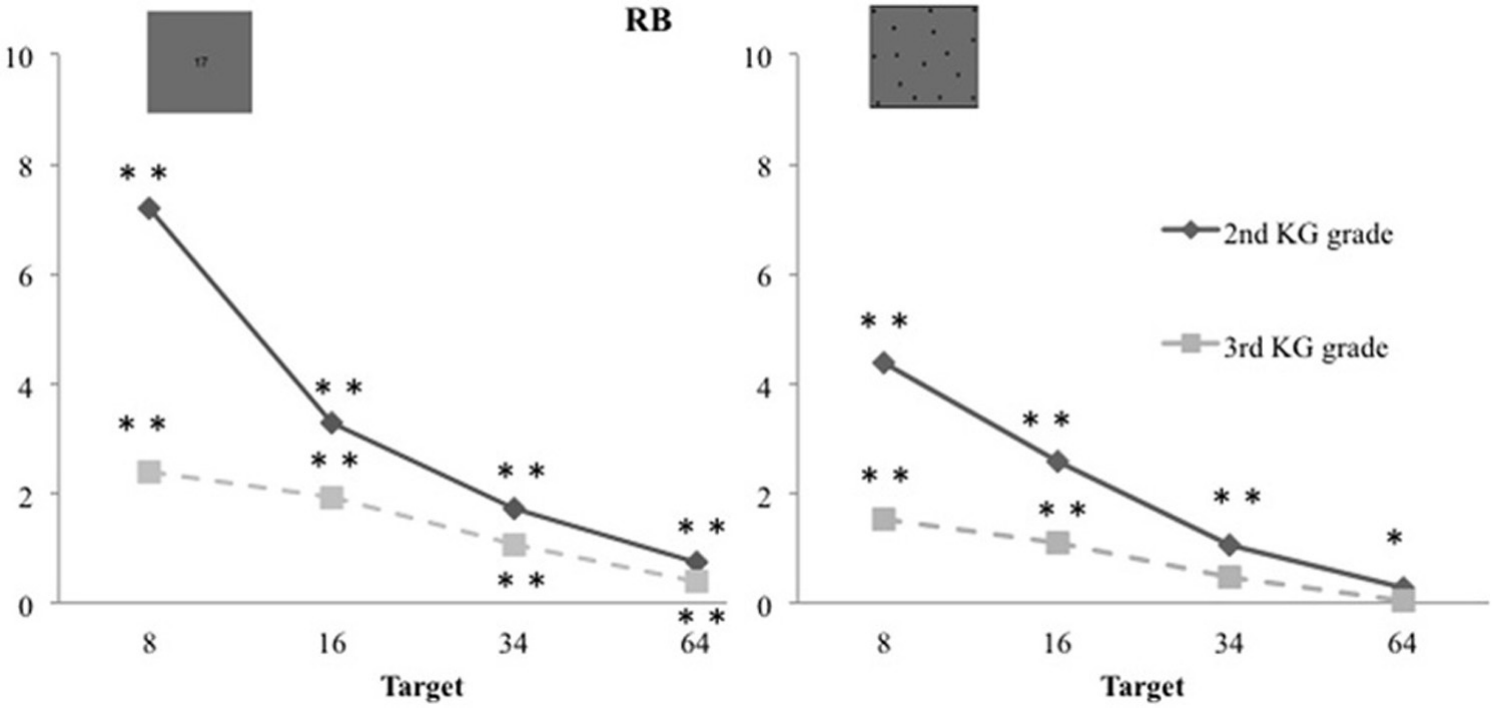
**Response-bias (RB) in the symbolic and non-symbolic tasks: children from 2nd and 3rd grade of Kindergarten overestimated the numerosity of the arrays**.

Children of both testing times (i.e., 2nd and 3rd grade of Kindergarten) overestimated the numerosity of the arrays (Figure 3). To describe this tendency we computed the response-bias [RB = (child's response – target magnitude)/target magnitude] and tested it against zero using *t*-tests. A RB of zero indicates that estimates were accurate, a negative RB that target magnitudes were underestimated and a positive RB that target magnitudes were overestimated. Contrarily to the expected underestimation predicted by the bi-directional mapping hypothesis (e.g., Castronovo and Seron, 2007), preschool children overestimated target magnitude in the symbolic “Dots to AN” task [2nd grade children RB: *M* = 3.237; *SD* = 3.002; *t*_(41)_ = 6.989, *p* < 0.001; 3rd grade children RB: *M* = 1.445; *SD* = 1.955; *t*_(31)_ = 4.182, *p* < 0.001]. They also overestimated in the non-symbolic estimation task [2nd grade children RB: *M* = 2.075; *SD* = 2.024; *t*_(41)_ = 6.645, *p* < 0.001; 3rd grade children RB: *M* = 0.786; *SD* = 1.359; *t*_(31)_ = 3.270, *p* = 0.003]. This positive RB was shown by the preschool children of the 2nd grade on every target magnitudes of both estimation tasks. Preschool children of the 3rd grade also overestimated numerosities of all target magnitudes in the symbolic “dots to AN” task. But in the non-symbolic task only the two smallest target magnitudes were overestimated (see Figure 3).

In summary, preschool children's stable COVs indicate that they were able to produce approximate estimates of large numerosities, which they consequently overestimated, as revealed by their positive response biases.

Whereas the COV measures how consistently children execute the estimation task with respect to the target numerosity, they do not inform about the precision of children's representations. A look at the mean estimates produced by the children indicates that their approximate non-symbolic estimations (“how many dots create a corresponding quantity”) as well as their symbolic estimations (“which AN describes a corresponding quantity”) were quite far from the expected target sizes. The following analyses will provide more information about children's absolute accuracy to perform symbolic and non-symbolic numerical estimation tasks.

#### The precision of estimates

In a second step the precision of children's numerical estimation was calculated as an absolute error score (AES) computed as follows: |participant's estimate answers—target magnitude|. The absolute value of the sum was provided as a measure of overall accuracy without paying attention to the direction of the difference between the target and the response.

***The effect of grade on the precision of estimates***. To evaluate the influence of early schooling (2nd and 3rd Kindergarten grade) on the precision of numerical estimation, an ANOVA on AESs was performed with target size (8, 16, 34, 64) and tasks (non-symbolic and symbolic estimations) as within-subject factors, and the testing times (2nd and 3rd Kindergarten grade) as the between-subjects factor. According to the previous analyses regarding the scalar variability of the estimates, the target size effect was significant, *F*_(3, 216)_ = 23.042, η^2^ = 0.242, *p* < 0.001, indicating that precision decreased with increasing target magnitudes. A significant effect of the task was also found, *F*_(1, 72)_ = 17.209, η^2^ = 0.193, *p* < 0.001, revealing that the non-symbolic estimation task (*M* = 35.643, *SD* = 31.26) led to higher accuracy (i.e., lower AESs) compared to the symbolic estimation task (*M* = 51.557, *SD* = 39.408). Finally, the time of testing effect was significant, *F*_(1, 72)_ = 9.875, η^2^ = 0.121, *p* = 0.002: 2nd grade preschool children were less accurate (*M* = 53.159, *SD* = 34.176) than 3rd grade children (*M* = 31,053, *SD* = 23,296). No other effect or interaction was significant.

***The effect of school's SEI on the precision of estimates***. To examine how important external factors such the socio-economic environment (here corresponding to the school's SEI) influence the precision of children's ANS, an ANOVA with the four target sizes, the two estimation tasks as within factors and the two levels of school' SEI as the between factor was performed for 2nd and 3rd grade children on AES (see also the descriptive information reported in Table 1B).

For the *2nd grade preschool children*, the target size effect was present, confirming the onset of typical approximate number representation characteristics as soon as 4–5 year olds, *F*_(3, 120)_ = 7.312, η^2^ = 0.155, *p* < 0.001. The effect of task was also significant, *F*_(1, 40)_ = 9.228, η^2^ = 0.187, *p* = 0.004. The non-symbolic estimation task gave rise to higher accuracy levels than the symbolic task (*M* = 44.507, *SD* = 35.177; *M* = 61.812, *SD* = 42.061, respectively). No other effect or interaction was significant.

Regarding the *3rd grade preschool children*, the target size effect was present, *F*_(3, 90)_ = 17.653, η^2^ = 0.370, *p* < 0.001, revealing the expected approximate number representation signature. The task effect was significant, *F*_(1, 30)_ = 9.047, η^2^ = 0.232, *p* = 0.005, showing again that 5–6 year old children are more accurate in the non-symbolic estimation condition (*M* = 24.008; *SD* = 20.463) compared to the symbolic one (*M* = 38.098; *SD* = 31.428). Most importantly, also SEI impacted their numerical representation significantly, *F*_(1, 30)_ = 4.410, η^2^ = 0.128, *p* = 0.044). Children from the low SEI school showed a less refined magnitude representation (*M* = 40.360; *SD* = 30.503) compared to children from the middle SEI school (*M* = 23.814; *SD* = 12.299).

**Figure 4:**
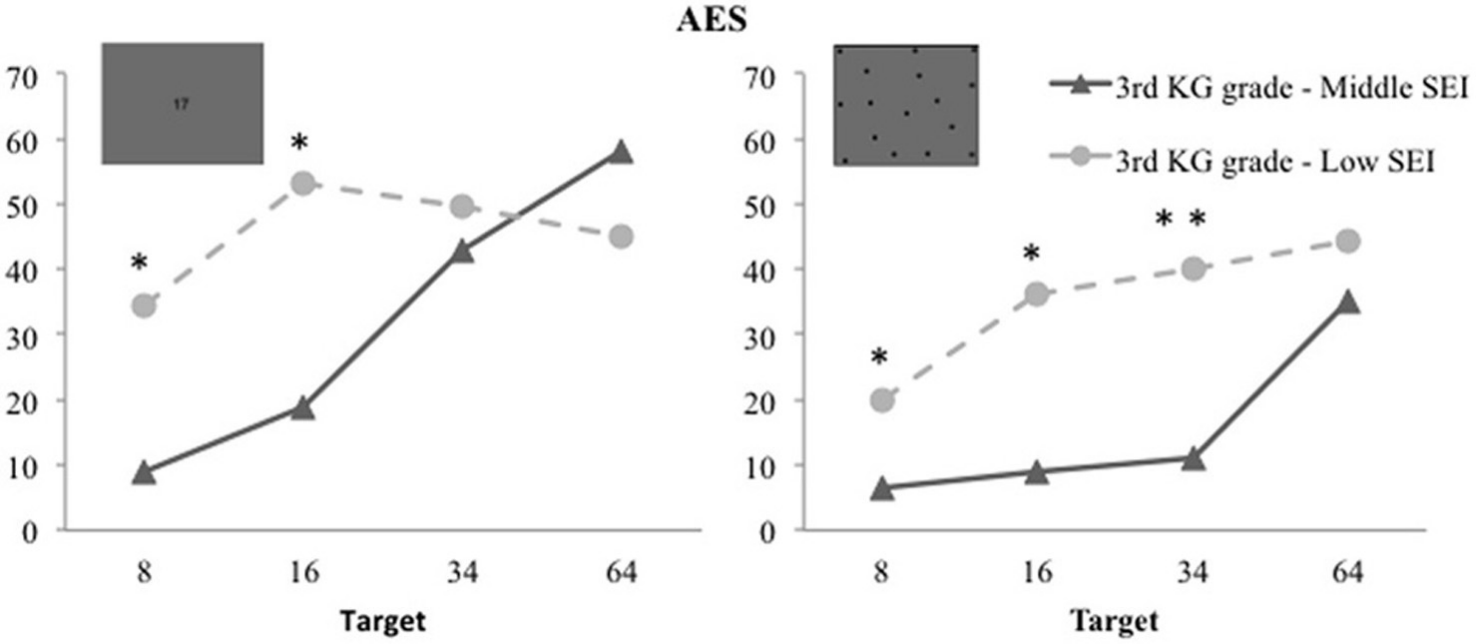
**Mean AES for low and middle SEI preschool children attending the 3rd grade, for each target magnitude in the symbolic and the non-symbolic estimation tasks.** Children from the low SEI school were significantly less accurate when estimating large numerosities. In the symbolic estimation task this SEI-related difference did not pertain to the two largest quantities. Note: ^*^Group differences significant at *p* < 0.05; ^**^ at *p* < 0.01.

In 3rd grade, significant double interactions between target magnitude and SEI, *F*_(3, 90)_ = 5.750, η^2^ = 0.161, *p* = 0.001, and triple interactions between target magnitude, SEI and task, *F*_(3, 90)_ = 3.241, η^2^ = 0.097, *p* = 0.026, were observed as well. The double interaction was due to the fact that middle SEI children were showing the expected effects of target magnitude increases on AES (the two tasks confounded, AES means were 7.694, 13.972, 27.074, 46.514 for targets 8, 16, 34, and 64, respectively) while low SEI children did not show this typical sign of approximate magnitude representation (27.185, 44.649, 44.9167, 44.691 for targets 8, 16, 34, and 64, respectively). Finally, the decomposition of the triple interaction by an ANOVA for each estimation task (4 target sizes × 2 SEIs) revealed that the 3rd grade low SEI preschool children had (i) poorer non-symbolic approximate representations [beside the Target size effect, *F*_(3, 90)_ = 11.076, η^2^ = 0.270, *p* < 0.001, the analyses showed a SEI effect, *F*_(1, 30)_ = 9.219, η^2^ = 0.235, *p* = 0.005]; (ii) and poorer symbolic estimation task performance over the small range of numerosities [beside the Target size effect, *F*_(3, 90)_ = 9.642, η^2^ = 0.243, *p* < 0.001, a Target size × SEI interaction was significant, *F*_(3, 90)_ = 6.053, η^2^ = 0.168, *p* = 0.001; see Figure 4].

In short, preschool children's estimates became less precise with increasing target magnitude, in line with the hypothesis that their response production relies on ANS recruitment. Moreover, 3rd grader's performances were generally more precise than that of their second grade peers. It was also in 3rd Kindergarten grade that the precision of children estimates were significantly influenced by their school's SEI (except for the production of large symbolic estimates).

### Early number competence tasks

In addition to measuring preschoolers' estimation abilities, we also assessed children's exact early number competences using number processing tasks involving numerosities up to 12 items. Descriptive information concerning means and standard deviations for the symbolic association and non-symbolic trade tasks for each group of children (according the period of the testing session and the SEI) are reported in Table 1C. Firstly, we investigated the influences of (a) the time of testing and (b) the school's SEI on children's performances in two exact numerical tasks. The first allowed us to assess the influence of schooling whereas the second evaluated the effect of socio-economic environment on children's early number competence. Accordingly, a repeated-measures analysis of variance (ANOVA) with the different testing times (2) and school SEIs (2) as the between-subjects factor were conducted regarding the two exact numerical tasks (the non-symbolic trade and the symbolic association tasks as within-subject factors). Results showed a significant effect of time of testing, *F*_(1, 70)_ = 67.974, η^2^ = 0.493, *p* < 0.001, with the preschool children of the 2nd grade (*M* = 5.488; *SD* = 2.686) reaching lower performance levels than the children of the 3rd grade (*M* = 10.140; *SD* = 2.017). The effect of SEI was also significant, *F*_(1, 70)_ = 5.140, η^2^ = 0.068, *p* = 0.026, showing that lower SEI participants (*M* = 6.721; *SD* = 3.322) were less efficient compared to the children from the middle SEI school (*M* = 8.163; *SD* = 3.253). No other effect or interaction was significant. Thus, preschoolers' understanding of the one-to-one correspondence principle (non-symbolic trade task), as well as their cardinal understanding of small Arabic digit symbols (symbolic association task) were directly influenced by (a) the level of Kindergarten schooling and (b) the SEI of the school that they were attending.

### Correlation between non-symbolic and symbolic number knowledge

In order to evaluate the relationship between preschool children's symbolic and non-symbolic exact numerical knowledge (i.e., based on their scores in the association and trade tasks, respectively) and their symbolic and non-symbolic approximate magnitude representations, correlation analyses were performed (Table 3).

**Table 3:** **Results of the Pearson correlations between the two exact numerical tasks (symbolic association and non-symbolic trade tasks) and the two estimation tasks (the non-symbolic and the symbolic one) performed by preschool children**.

			**Symbolic association task**	**Non-symbolic trade task**
3rd grade	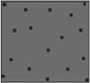	*r*	−0.529^**^	−0.437^*^
	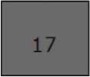	*r*	−0.382^*^	−0.208
2nd grade	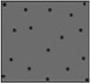	*r*	−0.252	−0.373^*^
	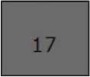	*r*	−0.153	−0.108

* Correlation significant at p < 0.05 (bilaterally);

** at p < 0.01 (bilaterally).

They revealed that, in 2nd and 3rd graders, the non-symbolic exact numerical task (i.e., the trade task), which assesses the non-verbal understanding of the one-to-one correspondence principles correlated with the non-symbolic estimation task. However, the entirely non-symbolic trade task did not correlate with the symbolic estimation task in either group.

Finally, the association task, which evaluated cardinal knowledge of number symbols up to 12, correlated with both symbolic and non-symbolic estimation of large quantities in 3rd graders only. This last result is congruent with the idea that children who present better exact symbolic knowledge are also those with the most refined ANS. However, this was true *only* for 3rd grade preschool children who were at a few months of receiving mathematics instructions. Indeed, no relation between the mastery of number symbols and estimation abilities could be found 1 year earlier in children attending 2nd Kindergarten grade.

## Discussion

To better understand approximate number processing and how it relates to exact number knowledge, the present paper explored young children's numerical abilities before they enter formal math education. To this aim we used a free estimate production paradigm and investigated for the first time preschooler's abilities to estimate large numerosities (ranging from 8 to 64 elements). Our data show that 5–6 year old children were able to produce estimates of large numerosities which reveal the typical ANS signature: Mean estimates and standard deviations both augmented constantly with increasing numerical target size. These results suggest that preschool children accessed the ANS in estimation tasks akin 9–10 year olds and adults in similar tasks (Castronovo and Göbel, 2012; Crollen and Seron, 2012; Mejias et al., 2012a,b).

By letting children freely produce estimates of several large numerosities we obtained direct insights into their ANS representations, in the sense that answers were not constrained by double choices (as typically the case in comparison tasks, e.g., Gilmore et al., 2007, 2010; De Smedt and Gilmore, 2011). Moreover, free estimation paradigms are more sensitive to individual differences than comparison tasks because they do not require the use of a limited number of numerosity ratios, i.e., difficulty levels (see for example Gilmore et al., 2010; De Smedt and Gilmore, 2011 vs. Halberda et al., 2008; Mussolin et al., 2012). Although preschoolers' estimates were relatively far from the targets, the stable COVs across targets (see Huntley-Fenner, 2001 for a detailed discussion of this measure) indicate that this was not due to a lack of compliance or an inability to perform the task. This performance pattern rather resulted from the fact that free estimation paradigms allow capturing more response nuances in a greater number of participants. Indeed, these paradigms also allow keeping the entire set of subjects for data analysis. This starkly contrasts with comparison studies, which tend to reject outliers (e.g., De Smedt and Gilmore, 2011; Sasanguie et al., 2013). However, especially these rejected participants could have highly informative analogue representations. In the present free estimation approach the data of all the tested preschool children were included in the analysis. Evaluating the stability of the COV across increasing target numerosities informed us on the consistency of preschoolers' responses while revealing their access to analog magnitude representations. Given the well-documented ANS acuity *increase* with age, the present COV averages (0.58 ± 0.13; 0.56 ± 0.17) from the symbolic and the non-symbolic tasks, respectively, are in line with the average COVs from the symbolic (0.36 ± 0.14) and non-symbolic (0.35 ± 0.12) tasks obtained in 9 year olds with a similar paradigm (Mejias et al., 2012a). Moreover, the difference in maximal estimation set size (20 vs. 64 here) explains why the 5 year olds in the study of Huntley-Fenner (2001) achieved a better COV of 0.37 than the children in the present study.

Contrary to the systematic underestimation bias observed in adults which perform approximate non-symbolic to symbolic mapping tasks (e.g., Castronovo and Seron, 2007), the young children of the present study overestimated the number of elements in the target sets. Although this result will need to be confirmed in future studies, it suggests a developmental trend from over- to underestimation. According to the bi-directional mapping hypothesis (Castronovo and Seron, 2007; Crollen et al., 2011; Crollen and Seron, 2012) adults are thought to underestimate large numerosities because they systematically map their logarithmically compressed approximate representation of the target set to its corresponding exact and linear representation of Arabic number symbolic (for an illustration, see Figure 2 in Crollen et al., 2011). Since preschoolers did not yet develop an exact linear representation of large number symbols (which will only be acquired in primary school), they need to rely on a more primitive and noisier symbolic representation of large numbers, resulting in systematic overestimation. The observation that preschool children performed better in the non-symbolic “dots to dots” task compared to the symbolic “dots to AN” task perfectly matches with this proposal. Combined with the overestimation, it indicates that young preschool children rely on qualitatively different estimation processes compared to older children and adults who received formal math education and perfectly master number symbols. Indeed, 9 year old typically achieving children as well as adults do not differ in non-symbolic vs. symbolic estimation tasks. Dyscalculic children, in contrast show the same profile than our pre-school children, that is a relatively better performance in the non-symbolic estimation task (Mejias et al., 2012a,b).

Although all tested preschooler populations relied on the ANS during approximate number processing, time of testing also had an impact on the ANS accuracy since 2nd grade preschool children were less precise compared to their 3rd grade peers in both symbolic and non-symbolic estimation tasks. In addition to instruction, the SEI of the school that children attended significantly influenced preschoolers' estimation performance, but only in 3rd grade of Kindergarten. This relatively late effect of the socio-economic status on the ANS precision contrasts with the fact that children's early number competence was already affected by school SEI in 2nd grade. The latter results confirm previous reports that 5 year old children from a low socio-economic environment have significantly worse early number skills compared to their socio-economically more advantaged peers at the end of Kindergarten (Jordan et al., 2006) and they extend them to 4 year olds (i.e., 2nd KG graders). The above-mentioned findings reinforce the proposal that the ANS is an innate system that is naturally predetermined to process numerosities and serves as a building block for the development of exact symbolic number competence (e.g., Barth et al., 2006). The newly learned number symbols then in turn positively influence the approximate numerical abilities. Within this theoretical framework the effects of SEI are expected to appear at different ages for exact and approximate number processing since external factors such as SEI will *first* affect the culturally acquired early number skills which will *then* through retro-influence refine the innate number sense.

In the present study, even preschool children were able to use complicated number symbols (>5 or even 10) to estimate numerosities in a non-random and meaningful way characterized by ANS signatures. However, in the youngest group these approximate symbolic abilities were completely independent of exact symbolic number. The latter link did, eventually, emerge in 3rd Kindergarten grade. Just as Sasanguie et al. (2013) observed with young primary school children, 3rd grade preschoolers who showed the best early number competence were also those who could process non-symbolic numerical magnitudes most precisely in the present study. The process of mapping exact symbolic knowledge onto the innate non-symbolic system thus seems to start even sooner than what has been observed by these researchers. This proposal fits with recent reports that young children's performance in non-symbolic comparison (Mussolin et al., 2012) and approximate addition (Gilmore et al., 2010) tasks significantly relates to their exact numerical abilities. It is, however, at odds with the predominant failure to find systematic associations between ANS and symbolic arithmetic learning (e.g., Piazza et al., 2010; Soltesz et al., 2010). As mentioned earlier, we propose that the positive findings with the present design might be due to the combination of several methodological parameters (i.e., no number-ratio restriction, high number of repetitions, no participant rejection), which optimize the paradigm's sensitivity to individual differences in ANS acuity. According to this interpretation we should also be able to observe this relation for primary school children (e.g., Holloway and Ansari, 2009; Soltesz et al., 2010; De Smedt and Gilmore, 2011) if sufficiently fine-grained assessment methods are used.

Whereas the symbolic estimation (i.e., “dots to AN”) only related to 3rd grade preschooler's early number competence, non-symbolic estimation (i.e., performance in the “dots to dots” task) was related to non-symbolic early number competence in both grades. Positive correlation between performance in the two types of non-symbolic tasks can easily be explained by the common low-level perception processes that underlie task performance and are present from an early age, independently of preschool education and the understanding of the verbal counting system (Brannon and Van de Walle, 2001; Rousselle et al., 2004). In contrast, the fact that symbolic estimation only related to early number competence at the end of Kindergarten indicates that the integration of approximate and exact representations of number symbols is emerging later in development. Combined with the (relatively) late influence of the school's SEI on estimation abilities, it suggests that the innate ANS might initially be impervious to external influences and further supports our hypothesis that newly learned exact symbolic number competences retro-influence ANS precision. Indeed, estimation abilities of 2nd grade preschoolers did not correlate with their early number competences^4^ (which depend on formal instruction), nor were they influenced by contextual factors (such as the school's SEI). From 2nd to 3rd Kindergarten grade estimation abilities then globally improved and achieved a higher maturational level, at which school SEI and pre-mathematical instruction interacted with estimation performances. This suggests that early ANS abilities are relatively insensitive to external factors (such as education and SEI) while maturating up to a certain developmental stage, here 3rd Kindergarten grade. Only once this maturational stage has been reached, the ANS is then systematically affected by the mastery of number symbols and SEI, amongst others. These developmental outcomes are similar to the results observed in adult Munduruku, since data on this Amazonian indigene group show that Munduruku with a certain level of symbolic number knowledge have a more refined ANS than their completely un-educated/instructed peers (Pica et al., 2004). They are also in line with a recent study showing that approximate number skills are less precise in western adults who did not received a formal math education that in their math-educated peers (Nys et al., 2013). Taken together, they support the idea that the ANS serves as cognitive scaffold for the development of the exact symbolic system, especially if future studies could highlight a developmental switch from over-toward underestimation, which is expected to occur when the exact symbolic number system has been acquired through instruction.

Finally it is worth noting that our study also provides insights into the directions that should be taken for developing optimal educational strategies. Taken at face-value the present results indeed suggest that early numeracy interventions should focus on developing good exact symbolic knowledge and then reinforce the link between the innate number sense and those learned symbolic skills.

## Conclusion

Our study addressed the relationship between approximate number processing and the exact number knowledge in Kindergarten children coming from different socio-economic environments. By investigating for the first time preschooler's abilities to estimate numerosities which largely exceed the numerical values they master exactly we found surprising estimation abilities at a very young age, i.e., from 5 years on.

Compared to their 2nd grade peers, children attending the 3rd grade of Kindergarten produced more accurate symbolic and non-symbolic estimates. In this group, which was close to entering primary school (and formal math education) we also observed a robust relationship between exact symbolic knowledge and ANS acuity. Moreover estimation abilities of 3rd grade Kindergarten children were influenced by the socio-economic context. This relatively late effect on ANS contrasted with the observation that SEI already influenced children's exact early number competence in 2nd Kindergarten grade.

Using a free estimation approach allowed us to disclose a link between the ANS and early number competences, which seems more difficult to highlight with other paradigms. Accordingly, we propose that this method is a very promising tool to obtain further direct insights into the characteristics and development of children's ANS representations.

### Conflict of interest statement

The authors declare that the research was conducted in the absence of any commercial or financial relationships that could be construed as a potential conflict of interest.

## Footnotes

^1^That is representations in the sense of first and second order isomorphisms (the structure of the representation contains information about the structure of the object that is represented and the relations that hold between external objects are supposed to exist in a similar fashion in the corresponding form of mental representations, e.g., Lass et al., 1993).

^2^In Belgium, by the end of the Kindergarten, children are familiarized and manipulated numbers until 6.

^3^The SEI of schools was established in Belgium in 1998 to allocate resources within the framework of the positive discrimination. This index is updated every five years and it is constructed from the variables “per capita income, educational attainment, unemployment, occupational and comfort level of housing.” To each student corresponds an index defined by its area of residence. It is the smallest administrative unit for which socioeconomic data are available. The SEI is then defined based on the average of the indices of its student population; it does not correspond directly to the area of implantation, or a measure of school performance. It allows one to rank schools on a scale of 1–20, from the lowest SEI to the highest. The choice of variables, indices and formula has been approved by the Government of the French Community (de Villers and Desagher, 2011).

^4^Except for the correlation between performances in non-symbolic tasks, due to shared perceptual processes.
